# Circulating circRNA: a social butterfly in tumors

**DOI:** 10.3389/fonc.2023.1203696

**Published:** 2023-07-21

**Authors:** Shuo Miao, Qingsong Zhang

**Affiliations:** ^1^Department of Urology, Affiliated Hospital of Qingdao University, Qingdao, China; ^2^School of Basic Medicine, Qingdao University, Qingdao, China

**Keywords:** circulating circRNAs, cancer, cell communication, diagnosis, therapy

## Abstract

Circular RNAs (circRNAs) are a class of single-stranded non-coding RNAs that form circular structures through irregular splicing or post-splicing events. CircRNAs are abnormally expressed in many cancers and regulate the occurrence and development of tumors. Circulating circRNAs are cell-free circRNAs present in peripheral blood, they are considered promising biomarkers due to their high stability. In recent years, more and more studies have revealed that circulating circRNAs participate in various cellular communication and regulate the occurrence and development of tumors, which involve many pathological processes such as tumorigenesis, tumor-related immunity, tumor angiogenesis, and tumor metastasis. Understanding the role of cell communication mediated by circulating circRNAs in tumor will further reveal the value and significance behind their use as biomarkers and potential therapeutic targets. In this review, we summarize the recent findings and provide an overview of the cell-cell communication mediated by circulating circRNAs, aiming to explore the role and application value of circulating circRNAs in tumors.

## The structure, regulation and function of circRNA

### Structure

Unlike linear RNAs terminated with 5’ caps and 3’ tails, circRNAs are characterized by covalently closed-loop structures with neither 5’ to 3’ polarity nor a polyadenylated tail. CircRNA is produced by pre-mRNA through back-splicing or alternative (back) splicing. No specific motifs are required for circularization except the splice sites (1, 2). A median exon length of 353 nucleotides is required for single-exon back-splicing, compared with only 112-130 nucleotides per exon for multiple-exon back-splicing in human cells (3). CircRNA can be divided into three types according to its composition: exon-derived circRNA (this type of circRNA is composed of single or multiple exons of a gene), intron-derived circRNA (consisting of a single intron, mainly derived from the lasso RNA and tRNA introns produced by pre-mRNA splicing) and exon−intron circRNA (including both exons and introns). Most endogenous human circRNAs contain several exons, usually two or three (3).

### Regulatory mechanisms of circRNA expression

Back-splicing and alternative splicing mediate the formation of circRNA. According to reports, intronic complementary elements facilitate back-splicing to increase circRNA levels (2). Exon circularization efficiency can be regulated by competition between RNA pairings across flanking introns or within individual introns. Importantly, the alternative formation of inverted repeated Alu pairs and the competition between them can lead to alternative circularization, resulting in multiple circular RNA transcripts produced from a single gene. In addition, RNA binding proteins (RBPs) regulate the formation of circRNAs. The one hand, RNA binding proteins (RBPs) directly bridge distal splice sites to promote back-splicing (4). On the other hand, RBPs bind to intronic complementary elements to enhance (5) or suppress (6) back-splicing by enhancing or reducing the pairing capacity of intronic complementary elements. It has also been reported that circular RNA biogenesis can proceed through an exon-containing lariat precursor without relying on cis elements or trans factors (7). Abnormal circRNA expression may also be associated with changes in genomic DNA (8).

### Functions of circRNA

The expression of circRNAs is cell-specific and developmental stage-specific (9, 10), and their biological functions are diverse. CircRNAs can negatively regulate pre-mRNA splicing, resulting in a reduction in linear mRNA levels and changing the composition of processed mRNA. Some circRNAs may be reverse transcribed to cDNA and incorporated into the genome. Stable circRNAs and EIcircRNAs are localized in the nucleus where they bind to the elongated RNA Pol II and promote transcription. They can act as sponges by competitively binding miRNAs to aid in miRNA transport or inhibit the binding of miRNAs to target genes (11). In addition, circRNAs can bind to RBPs and act as protein sponges (12). They can also bind two or more proteins to act as scaffolds and promote the interaction between enzymes and substrates (13). Besides, they can encode functional proteins (14).

## Circulating circRNA and cell-cell communication

​Circulating circulatory RNAs, as their name suggests, are cell-free circulatory RNAs present in peripheral blood. They mainly come from passive leakage of dead cells or active secretion of living cells (15, 16). They exist in two forms: free circRNAs that bind to proteins, and circRNAs in exosomes or microparticles. Compared to other circulating RNAs, circulating RNAs facilitate cell communication to a greater extent, reach a wider range of target cells, and have more opportunities for contact with other tissues and cells. These characteristics endow them with special advantages and important functions in cell-cell communication (**Table 1**).

**Table 1:** The communication between tumor cells and nontumor cells is mediated by circulating circRNAs.

Cancer	Circulating circRNA (source)	Mechanism	Clinical significance	Ref
CRC	hsa_circ_0136666(tumor cell)	Stimulate Treg cells by the miR-497/PD-L1 axis	Potential therapeutic target	(17)
	circPACRGL(N/A)	Increase the percentage of N2 neutrophils and promotes CRC proliferation and metastasis		(18)
Cholangio- carcinoma	Hsa_circ_0000284(tumor cell)	Regulate the behavior of tumor cells and induces malignant transformation of normal cells	Potential therapeutic target	(19)
	Circ_0020256 (TAM)	Promote the proliferation, migration, and invasion of cholangiocarcinoma cells via the miR-432-5p/E2F3 axis.		(20)
HCC	hsa_circ_0051443(liver cell)	Inhibit malignant biological behavior by competitively binding miR-331-3p to promote apoptosis and block the cell cycle.	Potential therapeutic target	(21)
	circRNA_DB (adipocyte)	Promote HCC growth and reduces DNA damage by inhibiting miR-34a and activating the USP7/Cyclin A2 signaling pathway		(22)
	circRNA-100338 (tumor cell)	Promote the proliferation of HUVECs and tube formation, regulates vasculogenic mimicry by regulating VE-cadherin.		(23)
	circ_4911, circ_4302 (tumor cell)	Promote the proliferation and migration of HUVECs		(24)
	CircMET (tumor cell)	Inhibit the infiltration of CD8+ T cells		(25)
	circGSE1 (tumor cell)	Promote HCC by inducing Treg expansion by regulating the miR-324-5p/TGFBR1/Smad3 axis		(26)
	circUHRF1 (tumor cell)	Inhibit NK cell-derived interferon-γ and TNF-α secretion by upregulating TIM-3 via degradation of miR-449c-5p		(27)
	hsa_circ_0074854 (tumor cell)	Induce macrophage M2 polarization, promoting the migration and invasion of HCC cells	Potential diagnostic and therapeutic targets	(28)
Glioma	circGLIS3 (tumor cell)	Stimulate the phosphorylation of ezrin (T567) in brain endothelial cells to promote angiogenesis	Potential diagnostic and therapeutic targets	(29)
	CircNEIL3 (tumor cell)	Recruit macrophages and enable them to acquire immunosuppressive properties.		(30)
	circRNA BTG2(macrophage)	Inhibit glioma progression via miR-25-3p/PTEN.		(31)
	has-circ-0015164, hsa-circ-0003243 (platelet)	N/A	Potential diagnostic and therapeutic targets	(32)
Pancreastic cancer	circ-IARS (tumor cell)	Increase Ras homolog gene family member A (RhoA) activity via absorption and regulation of miR-122, and the permeability of the endothelial monolayer is significantly enhanced.	Potential therapeutic target	(33)
Lung cancer	hsa-circRNA-002178 (tumor cell)	Induce PD1 expression in CD8+ T cells		(34)
	circZNF451 (tumor cell)	Induce an anti-inflammatory phenotype in macrophages, exhaustion of cytotoxic CD8+ T cells, and enhance TRIM56-mediated degradation of FXR1 to activate the ELF4-IRF4 pathway in macrophages	Potential biomarker and therapeutic target	(35)
	Circ-CPA4 (tumor cell)	Inactivate CD8+ T cells		
	circNDUFB2 (tumor cell)	Regulate the secretion of CXCL10, CXCL11, CCL5, and IFNβ, recruits CD8+ T cells and DCs into the tumor microenvironment		(36)
	circNRIP1 (platelet)	N/A	Potential biomarker and therapeutic target	(37)
OSCC	circKRT1 (tumor cell)	Work as a miR-495-3p sponge to regulate PD-L1 in CD8+ T cell.		(38)
	hsa_circ_0069313(tumor cell)	Promote Treg function by maintaining Foxp3 levels	Potential therapeutic target	(39)
BC	circ_002172 (tumor cell)	Inhibit CTL infiltration by upregulating CXCL12	Potential therapeutic target	(40)
	circ-TPGS2 (tumor)	Increase proinflammatory chemokine production and evoke tumor-associated inflammation by acting as a sponge of miR-7 and elevating TRAF6.		(41)
GC	circ_0008287(tumor cell)	Impair the function of CD8+ T cells and promote their apoptosis by binding to miR-548c-3 and increasing the expression of intracellular chloride channel protein 1.	Potential therapeutic target	(42)
BCa	circ_0001005 (tumor cell)	Sponge miR-200a-3p to promote PD-L1 expression, facilitating NK cell-mediated BCa cell killing.	Potential therapeutic target	(43)
	circDHTKD1 (tumor)	Recruit and activate neutrophils by inducing CXCL5 expression.		(44)
PCa	circSMARCC1(tumor cell)	Facilitate the expression of CD163 in macrophages through the CCL20-CCR6 axis and induce TAM infiltration and M2 polarization, leading to PCa progression.	Potential therapeutic target	(45)
Melanoma and lung cancer	mmu_circ_0000730	Involved in gut microbiota induced-cancer metastasis through the IL-11/circ_0000730/miRNA axis	Potential therapeutic target	(46)

### Communication between tumor cells

Cancer is a dynamic disease with high heterogeneity. Tumor heterogeneity may exist between individuals, tissues, sites, or cells, and contribute to the low efficacy or failure of therapies through the development of drug resistance. Tumor heterogeneity is derived from the genetic differences between cells and the differences in the microenvironment, and the information exchange between cells confers tumor plasticity and heterogeneity (47–49), as well as drug resistance. Cisplatin and 5-Fluorouracil (5-FU) are the most common anticancer drugs used for the treatment of a variety of solid tumors, such as ovarian cancer and lung cancer (LC), and adjuvant treatment of glioma (50–53). However, these drugs often result in the development of chemoresistance, leading to therapeutic failure. In esophageal cancer, circ_0000337-containing exosomes secreted by CDDP-resistant esophageal cancer cells could promote CDDP resistance in CDDP-sensitive esophageal cancer cells in vitro partly by regulating the miR-377-3p/JAK2 axis (54). Hon et al. studied the role of hsa-circ-0000338 in colorectal cancer (CRC) patients who were resistant to 5-FU and oxaliplatin (FOLFOX), they found exosomes transferred chemoresistance from FOLFOX-resistant HCT116-R cells into parental HCT116-P cells by selectively transferring hsa_circ_0000338 into recipient cells (55). Additionally, high expression of exosomal circ_0063526 in serum was associated with poor response to cisplatin treatment in gastric cancer (GC) patients. Exosomal circ_0063526 facilitated cisplatin resistance in GC by regulating the miR-449a/SHMT2 axis (56). Circulating circRNAs act as messengers between tumor cells, and targeting them would provide strategies for blocking intercellular communication and combating drug resistance.

In addition to drug resistance, the communication between tumor cells also involves symbiotic nutrient sharing, nutrient competition, and the transmission of oxidative stress (57). Reprogramming of energy metabolism is a hallmark of tumours caused by genomic instability. Recently, circRNAs have been reported to be associated with mutant glycolysis, lipogenesis and lipolysis, glutamate glycoside breakdown, and oxidative respiration in tumors (58). For example, silencing of circHIPK3, which is abundant in pancreatic islets, decreased Slc2a2 expression that encodes GLUT2 (59). CircHIPK3 could sponge miR-124, which represses the expression of several enzymes and transporters of glycolysis (60). However, it remains to be further investigated whether circRNAs are involved in metabolic regulation across different tumor cells.

### Communication between tumor cells and normal cells

The malignant transformation of normal cells is an important factor to promote the rapid progression of tumors. In cholangiocarcinoma, hsa_circ_0000284 was evidently elevated in plasma exosomes compared to normal controls, it enhanced the migration and proliferation of cholangiocarcinoma cells in vitro. Exosomes from cholangiocarcinoma cells stimulated the migration and proliferation of surrounding normal cells by transferring circ-0000284 (19). At the same time, circRNAs from normal cell-derived exosomes can be delivered to tumor cells. In hepatocellular carcinoma (HCC), hsa_circ_0051443 was packaged primarily in exosomes and transmitted from normal cells to HCC cells via exosomes, and it inhibited malignant biological behavior by competitively binding miR-331-3p to promote apoptosis and block the cell cycle (21). Hsa_circ_0051443 expression was significantly lower in plasma exosomes and tissues from patients than in those from healthy controls (21). This is a strategy for tumor cell survival, but it also provides lessons for treating tumors from normal cells.

### Communication between tumor cells and stromal cells

Stromal cells, together with extracellular matrix components, are critical components of the tumor microenvironment. Common stromal cells include fibroblasts, stellate cells, vascular endothelial cells, and adipocytes. The composition of stromal cells varies in different tumor tissues (61). The communication between stromal cells and tumor cells is the key to the dynamic shift of tumor microenvironment, which affects the occurrence and development of tumor.

### Fibroblasts

Cancer-associated fibroblasts (CAFs) constitute a major portion of the tumor stroma and play crucial roles in tumor progression and metastasis. CAFs derived either from resident fibroblasts or tumor-infiltrating mesenchymal stem cells (MSCs). When recruited into the tumor stroma, bone-marrow-derived MSCs can promote cancer stem cell development by secreting a specific set of paracrine factors or transforming into pro-stem cell CAFs. CircRNAs are essential mediators of the intercommunication between the tumor and CAFs, supporting tumor growth, survival, invasion and metastasis. For instance, hypoxia could induce secretion of exosomal circEIF3K from CAFs, and exosomal circEIF3K promoted colorectal cancer (CRC) progression via miR-214/PD-L1 axis (62). Hsa_circ_0056686, derived from CAFs, promoted cell proliferation and suppressed apoptosis in uterine leiomyoma through inhibiting endoplasmic reticulum stress (63). Moreover, breast cancer cells exposed to CAF-derived exosomes carrying circHIF1A display significantly increased stemness (64). In addition, there is evidence that circRNA is involved in the communication between tumor cells and MSCs. For example, exosomal circ_0030167 derived from bone-marrow-derived MSCs inhibited the invasion, migration, proliferation and stemness of pancreatic cancer cells by sponging miR-338-5p and targeting the Wif1/Wnt8/β-catenin axis (65). Tumor-associated exosomes play a significant role in the differentiation of fibroblasts into CAFs, as well as the differentiation of mesenchymal stem cells (66–68). Currently, there is evidence to show that circRNAs play a role in their differentiation (69–71), however, whether tumor-cell-derived circRNAs directly induce their differentiation still needs to be further revealed.

### Stellate cell

Stellate cells are resting stem cells of mesenchymal origin located in the liver and pancreas. Hepatic stellate cells (HSCs) comprise a minor cell population in the liver but serve numerous critical functions in hepatic physiology and pathology (72). HSCs are primarily known for their activation upon liver injury and for producing the collagen- abundant extracellular matrix in liver fibrosis (72). During HCC progression, activated HSCs are thought to accelerate carcinogenesis by affecting proliferation (73), migration and invasion (74), and angiogenesis (75). Recently, several studies have revealed that circRNAs are involved in the communication between HCC cells and HSCs. For example, HSC exosome-derived circWDR25 promoted the progression of HCC via the miRNA-4474-3P-ALOX-15 and EMT axes (76). In addition, peritumoral circWDR25 secreted by HSCs affects the prognosis of HCC patients after radical hepatectomy, and their elevated expression in the adjacent tissues was closely related to a poor prognosis of the patients (77). In pancreatic cancer, upregulated circRNA chr7:154954255-154998784+ in cancer-associated pancreatic stellate cells promoted the growth and metastasis of pancreatic cancer by targeting the miR-4459/KIAA0513 axis (78). There are different subgroups of HSCs, and the subgroups have different roles in HCC (79). Whether circRNAs are involved in the activation of HSCs and the dynamical shift of HSC subpopulations still needs to be further revealed.

### Endothelial cells

Endothelial cells are involved in angiogenesis and tumor metastasis. Huang et al. revealed that circRNA-100338 helped mediate the communication between HCC cells and human umbilical vein endothelial cells (HUVECs) via exosomes (23). On the one hand, exosomal circRNA-100338 promotes the proliferation of HUVECs and tube formation. On the other hand, exosomal circRNA-100338 regulates vasculogenic mimicry by modulating VE-cadherin. In glioma, circGLIS3 could be packed into exosomes and absorbed by human brain microvascular endothelial cells, stimulating the phosphorylation of ezrin (T567) to promote angiogenesis (29). During tumor metastasis, changes in endothelial permeability act as accelerators for tumor expansion. Circulating circRNAs act as the “key” that allows tumor cells to “open” the endothelial cell barrier. In pancreatic cancer, circ-IARS expression is upregulated in cancerous tissues and in plasma exosomes of patients with metastatic disease. Circ-IARS was found to enter HUVECs through exosomes, it significantly increased Ras homolog gene family member A activity via absorption and regulation of miR-122, and the permeability of the endothelial monolayer was significantly enhanced (33). It was reported that increased Ras homolog gene family member A activity in HUVECs promoted actin-cytoskeletal remodeling and cell contraction and reduced the expression of the tight junction ligand protein Zonula occludens-1, leading to endothelial barrier dysfunction (80, 81) and endothelial hyperpermeability (82, 83). Cell migration is driven by local membrane protrusion through directed polymerization of F-actin at the front (84). Circ-IARS also increased F-actin expression and focal adhesion in HUVECs (33).

Besides, pericytes are also one of the main cellular components, they are typically described as greatly elongated, slender, and branched cells, with projections that extend longitudinally and circumferentially around the vessel wall (85, 86). They support the formation and function of blood vessel, and can be recruited during tumor angiogenesis (87, 88). However, the mechanism by which they are recruited is unclear. Whether it involves communication between tumor cells and pericytes, endothelial cells and pericytes remains to be explored further.

### Adipocyte

​Adipocytes are specialized cells that regulate energy balance, store excess energy as fat, and play a regulatory role in tumors by secreting metabolites, enzymes, hormones, growth factors, and cytokines. During the development of cancer, tumor cells have a metabolic symbiosis with neighboring adipose tissue. For example, adipocytes provided adipokines and lipids to cancer cells and regulated therapeutic resistance (89). Zhang et al. reported that circRNAs secreted by adipocytes regulated the development of HCC. Exosomal circRNA_DB was upregulated in HCC patients with high body fat ratios. CircRNA_DB secreted by adipocytes promoted HCC growth and reduced DNA damage by inhibiting miR-34a and activating the USP7/Cyclin A2 signaling pathway (22). Increased adiposity contributes to carcinogenesis and tumor progression, but advanced stages of numerous cancers are associated with loss of white adipose tissue and wasting of the body, which complicates treatment and adversely affects patient survival. Exploring the communication between adipocytes and tumor cells may provide an answer to this question.

### Communication between tumor cells and immune cells

#### T cells

Current immunotherapeutic methods mainly focus on T lymphocytes, especially restoring exhausted cytotoxic T cells (CTLs). An example of such an approach is immune-checkpoint blockade, in which monoclonal neutralizing antibodies block of receptors or ligands that inhibit the activation of CTLs, including programmed cell death protein 1 (PD1), PD1 ligand PD-L1 and lymphocyte-activation gene-3 (90). The use of PD1 antibodies decreases tumor progression and provides long-term clinical benefits in patients (91, 92). However, most patients inevitably acquire resistance after several cycles of treatment (93). More and more mechanisms have been revealed, including the role of circulating circulating RNA.

First, circulating circRNAs are important regulators of PD1 expression in CTLs. In lung adenocarcinoma (LUAD), hsa-circRNA-002178 was significantly upregulated in LUAD tissues and LUAD cancer cells. It can be detected in the exosome of plasma from LUAD patients and could serve as a biomarker for early diagnosis of LUAD. Hsa-circRNA-002178 could enhance PD-L1 expression by sponging miR-34 in cancer cells, and circRNA-002178 could be delivered into CD8^+^ T cells to induce PD1 expression via exosomes (34). Second, circulating circRNAs inactivate CTLs by up-regulating PD-L1 expression in tumor cells. By coculturing non-small cell lung cancer (NSCLC) cells with CD8^+^ T cells isolated from human peripheral blood mononuclear cells in a transwell coculturing system, Hong et al. found that Circ-CPA4 positively regulated exosomal PD-L1. NSCLC cells inactivated CD8^+^ T cells in a secreted PD-L1-dependent manner, and NSCLC cells with circ-CPA4 ablation reactivated CD8^+^ T cells in the coculturing system (94). Third, circulating circRNAs regulate T cell penetration. In HCC, high levels of circMET were significantly correlated with a low density of tumor-infiltrating CD8^+^ lymphocytes. CircMET promoted HCC development by inducing EMT and enhancing the immunosuppressive tumor microenvironment through the miR-30-5p/Snail/dipeptidyl peptidase 4 (DPP4)/CXCL10 axis (25). In NSCLC, circNDUFB2 regulated the secretion of CXCL10, CXCL11, CCL5, and IFNβ, it is recognized by RIG-I to activate RIG-I-MAVS signaling cascades and recruited CD8^+^ T cells and dendritic cells into the tumor microenvironment, and circNDUFB2 downregulation in NSCLC tissues was correlated with NSCLC malignant features (36). What’s more, circulating circRNAs regulate T cell apoptosis. Upregulation of circ_0008287 in GC impaired the function of CD8^+^ T cells and promoted their apoptosis by competitively binding to miR-548c-3 and increasing the expression of intracellular chloride channel protein 1 (42).

The adaptive immune system is modulated by an essential subset of CD4^+^ T lymphocytes called regulatory T cells (Tregs) that function in maintaining immune homeostasis by preventing excessive immune activation. In tumors, Tregs secrete immunosuppressive factors to inhibit the function of CD8^+^ T cells, causing tumor immune escape (26, 95). In OSCC, hsa_circ_0069313 was upregulated and predicts a poor7 prognosis. It is an exosomal circRNA, and the transfer of hsa_circ_0069313 to Treg cells promoted Treg function by maintaining Foxp3 levels (39). In conclusion, circulating circRNA affects the body’s immunity by regulating the function of T cells in various ways, and targeting circulating circRNA will provide a new strategy for tumor immunotherapy.

#### Natural killer cells

In recent years, the rapid and potent antitumor function of innate immunity, which occurs even at highly early stages of tumor progression, has attracted increasing attention. As a subset of innate lymphoid cells, natural killer (NK) cells, commonly considered type 1 innate-like cells, are currently defined as effector cells, exerting natural cytotoxicity against primary tumor cells and metastasis by inhibiting migration and colonization to distant tissues (96). They can distinguish abnormal cells from healthy cells, leading to more specific antitumor cytotoxicity and reduced off-target complications (97). In addition, they can produce cytokines, mainly interferon-γ, to modulate adaptive immune responses and participate in other related pathways (98). Recently, NK cell dysfunction has been demonstrated in various malignancies. Several studies have shed light on the role and mechanisms of circulating circRNAs in NK cell dysfunction. On the one hand, circulating circRNA levels are correlated with the number of NK cells and their penetration into tumor tissue. In plasma from HCC patients, circUHRF1 was predominantly secreted by HCC cells in an exosomal fashion. Elevated plasma exosome circulating UHRF1 levels were associated with a decreased NK cell fraction and decreased NK cell tumor infiltration. CircUHRF1 inhibited NK cell-derived interferon-γ and TNF-α secretion by upregulating TIM-3 via degradation of miR-449c-5p, thereby promoting immune evasion (27). On the other hand, circulating circRNA modulates the killing effect of NK cells on tumor cells. For example, in bladder cancer (BCa), androgen receptor (AR) influenced the initiation and progression of tumors. Both androgen therapy and AR knockdown effectively reduced PD-L1 expression, facilitating NK cell-mediated BCa cell killing. Mechanistically, androgen receptor upregulated circ_0001005 expression via the RNA-editing gene ADAR2. Circ_0001005 competitively sponged miR-200a-3p to promote PD-L1 expression (43). In addition, the abnormal expression of circRNAs also regulated the toxicity of NK cells to tumor cells in HCC (99), breast cancer (100), and renal cell carcinoma (101).

#### Macrophages

Macrophages include many cell types with complex and delicate regulatory networks. The type, density and location of macrophages have good prognostic value in various cancer types. Tumor-associated macrophages (TAMs), including both resident macrophages and circulating monocytes recruited to the tumor microenvironment, are a predominant cell type in tumors (102). Under the guidance of different microenvironmental signals, macrophages differentiate into two functional phenotypes, namely, classically activated macrophages (M1) and alternately activated macrophages (M2). In contrast to the anti-tumor effects of M1 macrophages, M2 macrophages have anti-inflammatory and tumorigenic properties. Consistent with macrophages, TAMs are also highly plastic and switch from one phenotype to another (103, 104).

CircRNAs mediate macrophage infiltration, facilitating carcinogenesis and cancer development. CircNEIL3 derived from NEIL3 increased with increasing glioma grade and was regulated by EWS RNA-binding protein 1 (EWSR1). Functionally, circNEIL3 promoted tumorigenesis and progression of gliomas *in vitro* and *in vivo*. Mechanically, circNEIL3 stabilized IGF2BP3, a known oncogen, by preventing HECTD4-mediated ubiquitination. CircNEIL3-overexpressing glioma cells drove macrophage penetration into the tumor microenvironment by activating YAP1 signaling and secreting CCL2 and LOX. Moreover, circNEIL3 could be packaged into exosomes by hnRNPA2B1 and transmitted to infiltrating TAMs, thereby enabling them to acquire immunosuppressive properties by stabilizing IGF2BP3, in turn promoting glioma progression (30). In prostate cancer (PCa), high expression of circSMARCC1 was positively associated with colonization of CD68^+^/CD163^+^/CD206^+^ TAMs in the tumor microenvironment. Overexpression of circSMARCC1 facilitated the expression of CD163 in macrophages through the CCL20-CCR6 axis and induces TAM infiltration and M2 polarization, thereby leading to PCa progression (45). CircRNAs are also involved in M_2_ macrophage polarization. CRITGB6, which was robustly upregulated in tumor tissue and sera from platinum-resistant OC patients, was associated with a poorer prognosis. CircITGB6 overexpression promoted M2 macrophage-dependent CDDP resistance both *in vivo* and *in vitro*. Mechanistic research determined that circITGB6 directly interacted with IGF2BP2 and FGF9 mRNA to form a circITGB6/IGF2BP2/FGF9 RNA−protein ternary complex in the cytoplasm, thereby stabilizing FGF9 mRNA and inducing polarization of TAMs toward the M2 phenotype. Importantly, blocking M2 macrophage polarization with an antisense oligonucleotide targeting circITGB6 markedly reversed the circITGB6-induced CDDP resistance of OC *in vivo* (105). In HCC, hsa_circ_0074854 can be transferred from HCC cells to macrophages via the exosomes. Exosomes with downregulated hsa_circ_0074854 suppressed macrophage M2 polarization, which in turn suppressed the migration and invasion of HCC cells both *in vitro* and *in vivo* (106). Interestingly, the exosomal circRNAs produced by TAMs also affect tumors. Circ_0020256 in TAM-secreted exosomes promoted the proliferation, migration, and invasion of cholangiocarcinoma cells via the miR-432-5p/E2F3 axis (20). In HCC, exosomal miR-628-5p from M1-polarized macrophages hindered N6-methyladenosine (m^6^A) modification of circFUT8 to suppress HCC progression (107). In summary, circulating circRNAs affect tumor development by modulating the interaction between macrophages and tumor cells. Targeting circRNA-mediated information exchange between macrophages and tumor cells may be one of the strategies to reshape the tumor microenvironment.

#### Neutrophils

The role and importance of neutrophils in cancer has received increasing attention over the past decade. Many patients with advanced cancer show elevated neutrophilia levels. Intratumoural neutrophils (also known as tumor-associated neutrophils (TANs)) are connected to a dismal prognosis, and the neutrophil-to-lymphocyte ratio has been introduced as a significant prognostic factor for survival in many types of cancer (108). TANs constitute an important portion of the infiltrating immune cells in the tumor microenvironment, and they are recruited to the tumor and can acquire either protumor or antitumor functions (109). In BCa, circDHTKD1 was positively associated with lymph node metastasis and significantly upregulated. CircDHTKD1 interacted directly with miR-149-5p and antagonized CXCL5 inhibition by miR-149-5p. CircDHTKD1-induced CXCL5 expression recruited and activated neutrophils, which participated in lymphangiogenesis by secreting VEGF-C (44). Like TAMs, TANs are also divided into two types, N1 and N2 (37). N1 TANs have cytotoxic and antitumor effects, and N2 TANs promote tumor progression (110). Transforming growth factor-beta (TGF-β) is a multifunctional cytokine implicated in tumor initiation, progression, and metastasis. TGF-β promotes the formation of TANs. In particularly, TGF-β inhibits N1 but promotes N2 neutrophil differentiation (111). It has been reported that circPACRGL served as a sponge for miR-142-3p/miR-506-3p to facilitate TGF-β1 expression in CRC. CRC-derived exosomal circPACRGL increased the percentage of N2 neutrophils and promotes CRC proliferation and metastasis (18).

### Communication between tumor cells and platelets

Platelets participate in physiologic hemostasis and pathologic thrombus, and the role in tumors has also attracted considerable attention. They affect all aspects of cancer progression, most notably tumor cell metastasis. Platelets isolated from cancer patients frequently show different RNA and protein profiles without significant changes in hemostatic activity. This phenotype is unique to a population known as tumor-educated platelets. At present, the mechanism by which tumor cells educate platelets is not completely understood, but many studies have revealed the interaction between tumor cells and platelets. For example, platelets can interact with circulating tumor cells through receptors and ligands (112, 113), and platelets can be recruited to the tumor tissue microenvironment to play a regulatory role (114, 115). Of concern, platelets can directly take up and store RNAs (mRNAs, miRNAs, LncRNAs) from tumor cells, and tumor-educated platelets in liquid biopsies have emerged as valuable resources that can be used to assess cancer-related RNA profiles with tumor specificity, high sensitivity, and high accuracy (116–118). CircRNA levels in human platelets are 17- to 188-fold higher than those in nucleated tissues and 14- to 26-fold higher than those in samples digested with RNase R (119). Like other RNAs, platelet circRNAs have shown promising applications in human disease. For example, NRIP1 circRNA was identified to be pregnancy specific, with significant upregulation in maternal platelets in the first trimester compared to those from non-pregnant control participants. This finding allows NRIP1 circRNA to be used as a first-tier check (gold standard) in future efforts for diagnostic screening purposes using circRNAs as targets and maternal first-trimester platelets as a source (120). In NSCLC, 4732 circRNAs were detected in platelet samples from patients and controls, and 411 of these circRNAs were significantly differentially expressed; circNRIP1 is one representative of the differentially expressed circRNA. CircNRIP1, which was downregulated in the NSCLC group, could be employed as a potential biomarker for NSCLC (37). Chen et al. analyzed the platelet RNA profiles of 8 glioblastoma samples and 12 normal samples and constructed a ceRNA network (circRNA-mRNA−miRNA) based on 2 miRNAs (hsa-let-7a-5p, hsa-miR-1-3p), 2 mRNAs (CCR7 and FAM102A), and 2 circRNAs (has-circ-0015164, hsa-circ-0003243) to investigate the potential interactions (32). This study also sheds light on the potential relationship between platelet circRNAs and cancer, as well as the clinical value of platelet circRNAs. However, the cause of changes in platelet circRNA in patients with tumors remains unclear. These changes may be related to the uptake of circulating circRNA or may be the result of platelet-selective release (119). In addition, platelets are fragments from megakaryocytes, and tumor cells can also educate megakaryocytes (121). Whether these changes are the result of megakaryocytes being educated by tumor cells remains to be further revealed. What’s more, tumor-associated thrombosis is one of the common complications in tumor patients and is an important cause of death (122, 123), which is closely related to the abnormality of platelet quantity and function (124–126). Whether circulating circRNA is involved in platelet dysfunction remains to be further investigated.

### Communication between tumor cells and erythrocytes

Erythrocytes account for ∼84% of the total blood cells count in the average adult (127). Recently, several studies show that abnormal red blood cell distribution width is associated with poor prognosis in cancers (128, 129). Erythrocytes can interact with tumor cells (130), and erythrocytes from cancer patients have a differential proteome profile compared with cancer-free controls (131). In fact, erythrocytes also have high circRNA content besides platelets (132–134). Recently, Nicolet et al. provided the first detailed analysis of circRNA expression during erythroid differentiation (135). They found that circRNA expression not only significantly increased upon enucleation but also had limited overlap between progenitor cells and mature cells, and only one out of 2531 (0.04%) circRNAs was associated with mRNA translation regulation. These results suggest that circRNA expression originates from regulatory processes rather than from mere accumulation, yet their contribution to regulatory cellular processes is still unknown. Interestingly, the levels of approximately 4% circRNAs in the erythrocytes did not overlap with the levels of mRNAs in differentiated erythrocytes. These circRNAs may be acquired from other cell types in the circulation or endogenous vascular-derived RNA (136). If this is the case, circRNAs taken up by erythrocytes could be novel biomarkers for tumors due to their high stability. In addition, tumor-associated anemia (137), is the key reason for leading to a decline in patients’ quality of life. And anemia in tumor patients is associated with a decrease in the number of red blood cells (138). Whether circulating circRNAs are involved in the reduction of red blood cells is also an urgent question to be explored.

### Communication between tumor cells and the gut microbiota

The human gut harbors diverse microbes that play a fundamental role in the well-being of their host, including participating in energy collection and storage, fermenting and absorbing undigested carbohydrates (139), promoting the maturation of immune cells and the normal development of immune function (140). However, the influence of gut microbiota is not limited to the local part of the intestine but is instead systemic (141, 142). In recent years, the role of the gut microbiota in tumor-related immune regulation has attracted much attention. In particular, the gut microbiota regulates antitumor immunity through metabolites, which are small molecules that can be transported in the body and act on local and systemic antitumor immune responses to promote the efficacy of immune checkpoint inhibitor (ICI) therapy (143–146). Recently, Zhu et al. found that the levels of circulating miRNAs and circRNAs changed significantly with dysbiosis of the microbiota, and the gut microbiota regulated tumor metastasis via circRNA/miRNA networks. Specifically, the gut microbiota downregulated circulating mmu_circ_0000730 and upregulated circulating mmu-miR-466i-3p or mmu-miR-466 f-3p. SRY-box transcription factor 9 (SOX9) is a target gene of mmu-miR-466i-3p and mmu-miR-466 f-3p. Mmu_circ_0000730 upregulated SRY-box transcription factor 9 (SOX9) and activated epithelial-mesenchymal transition markers in cancer cells by targeting mmu-miR-466i-3p or mmu-miR-466 f-3p. Furthermore, intestinal flora reconstruction significantly decreased IL-11 expression. IL-11 treatment induced mmu_circ_0000730/SOX9 expression, while it downregulated mmu-miR-466i-3p and mmu-miR-466 f-3p. In cancer cells, IL-11 promoted SOX9 expression, induced cell invasion, and promoted the stemness of cancer stem cells (46). This study shows that there is a mechanistic link between the gut microbiota and cancer metastasis through the IL-11/circRNA/miRNA axis, which will help to pave the way to the clinical use of gut microbiota for cancer prevention or treatment in the future (147–149).

## Clinical applications of circulating circRNAs involved in cell-cell communication

### Biomarkers for tumors

Seeking specific molecules of tumor origin to improve the accuracy and sensitivity of early screening and prognosis assessment has consistently been an urgent issue. CircRNAs have attracted much attention in tumor biomarker studies due to their stability, tissue specificity of expression, and relatively high content (150). Circulating circRNAs are widely respected in the field of non-invasive liquid biopsy. Large numbers of clinical samples also reveal its huge application prospect (151–154). For example, AR is the key driver gene and a common target for the treatment of PCa. CircRNA AR3,derived from AR, is widely expressed in PC cells and prostate tumors. Plasma circRNA_AR3 level was positively associated with Gleason scores and lymph node metastasis of PCa, and it was undetectable in men after radical prostatectomy (151). It is considered a promising circulating RNA with tumor specificity. In fact, the circRNAs identified so far, tumor-specific circRNAs are very rare, and most of them are non-specific. However, these abnormally expressed circRNAs still have high diagnostic value (155–157). For example, in CRC, circRNA_PNN was found to have significant value for CRC diagnosis among 122 differentially expressed circRNAs. The area under the receiver operating characteristic curve of serum exosomal circ-PNN for early-stage CRC was 0.854 (sensitivity=91.7%, specificity=69.0%) (152). In addition, another study showed that exosomal HSA-circ-0004771 levels in serum were 14 times higher in patients with CRC than in healthy controls. Its level was correlated with stage and distant metastasis. The sensitivity and specificity values of exosomal hsa-circ-0004771 for differentiating CRC patients from HCs were 80.91% and 82.86%, respectively (153). To further improve diagnostic accuracy, several studies have analyzed multiple circulating circRNAs in combination, which performed well in tumor detection and showed a higher accuracy (158). For example, a plasma circRNA panel (CircPanel) (hsa_circ_0000976, hsa_circ_0007750 and hsa_circ_0139897) showed a higher accuracy than alpha-fetoprotein (AFP) in distinguishing individuals with HCC from controls. The circPanel also performed well in detecting small HCC (solitary, ≤3 cm), AFP-negative HCC and AFP-negative small HCC (158). These results indicate the high sensitivity and accuracy of circulating circRNAs in tumor diagnosis. The biological functions of these non-tumor specific circRNAs are not restricted to the tumor cells. Compared with tumor-specific circRNAs, those that promote malignant transformation of normal cells are also worthy of attention (19), they are the starting point of evil and the key point of evil progress. Moreover, angiogenesis is a prerequisite for rapid tumor development and a key to metastasis. Endothelial cell proliferation and angiogenesis based on the regulation of circulating circRNAs is another concern (24, 29), and the levels of these circulating circRNAs will provide a strong reference for evaluating tumor metastasis or prognosis.

### Biomarkers for tumor immunity

Compared with normal people, numerous tumor patients have obvious abnormalities in peripheral blood immune cells, including changes in number and function. In addition to the self-regulation of immune cells, the role of circulating circRNA should not be ignored. First, circulating circRNAs reflect the functional state of T cells. The functional status of CD8+T cells is of primary concern in current immunotherapy. Abnormally expressed circulating circRNAs cause T cell dysfunction in several ways, such as affecting PD1/PDL1 expression (25, 34, 94) or reducing infiltration (34, 159) or promoting their exhaustion (25, 40). In addition, circulating circRNA enhances CD8^+^ T cytotoxicity (38). The detection of specific circulating circRNAs will provide reference and support for the analysis of T cell functional status. Second, circulating circRNAs reveal the function of NK cells. NK cells are known for their cytotoxic role, but they are dysfunctional in tumors. On the one hand, NK cell infiltration is regulated by circRNA. A study involving 240 patients with HCC reported that there was a negative correlation between plasma circUHRF1 levels and the proportion of NK cells. With higher expression of circUHRF1, NK cell penetration also decreased. CircUHRF1 inhibited NK cell function by reducing the expression of interferon-γ and TNF-α, which promoted immune evasion (27). On the other hand, the killing effect of NK cells is regulated by circRNAs. Circ_0001005 competed with miR-200a-3p in sponging to promote PD-L1 expression. and NK cell-mediated BCa cell killing could be facilitated by downregulating circ_0001005 (43). Third, cancer-associated systemic inflammation is frequently characterized by a high ratio of neutrophils to lymphocytes and is associated with a poor prognosis, it occurs in both the late stages of the tumor and in the early stages (stage I and stage II, separately) before treatment (160–162). Exosomal circRNAs are involved in recruitment and activation of neutrophils (18, 44), suggesting their potential value as biomarkers. Therefore, the screening and identification of circulating circRNAs involved in cell communication could be useful for the detection of tumor-related immunity and would also provide a reference for the early prevention and treatment of tumors.

### Targets for therapy

In addition to acting as biomarkers, circulating circRNAs are promising therapeutic targets (163–166). For example, the expression of hsa_circ_0014235 was notably elevated in NSCLC serum derived exosomes. Hsa_circ_0014235 promoted proliferation and invasion of NSCLC cells. Hsa_circ_0014235 triggered the malignant development of NSCLC through the miR-520a-5p/CDK4 regulatory axis (163). In addition to the direct regulatory effect on tumor cells, the effect of circulating circRNA on other cells in the tumor background is also of interest. For example, in HCC, the exosomal circRNA-100338 enhanced communication between HCC cells and endothelial cells. It promoted the proliferation and tube formation of HUVECs and regulated vasculogenic mimicry (23). And circRNA_DB secreted by adipocytes promoted HCC growth and reduced DNA damage by inhibiting miR-34a and activating the USP7/Cyclin A2 signaling pathway (22). However, when exosomal circ_0051443 was transferred from hepatocytes to HCC cells, it inhibited HCC progression by promoting apoptosis and blocking the cell cycle (21). These studies further demonstrate that tumor development is a networked regulatory process based on multicellular communication, and that breaking cellular communication is key to blocking its development.

### Signals of drug resistance

Medication is critical, especially for patients who miss the best time for surgery. However, drug resistance significantly reduces the effectiveness of treatment and increases mortality. Circulating circRNAs are deeply involved in resistance to antitumor drugs, from traditional chemotherapeutic drugs to targeted and immunotherapeutic drugs (167–170). Numerous experiments have demonstrated that drug resistance was never the sole event of individual cells (55, 171). For example, in CRC, exosomes from FOLFOX-resistant CRC cells selectively transferred hsa_circ_0000338 into recipient cells to confer chemoresistance (55). The transmissibility of chemotherapeutic drug resistance is due to the heterogeneity and communication among tumor cells. Communication between tumor cells and immune cells is closely related to tumor immunotherapy resistance. Immunotherapy, represented by anti-PD-1/PD-L1 antibodies, has shown great efficacy in the clinical treatment of cancers (172). Circulating circRNAs are involved in immunotherapeutic resistance through mediated dysregulation of PD-1/PD-L1 expression (34, 38). Additional exploration of the role of circulating circRNA on immune cells will shed further light on the mechanism of resistance.

### Potential challenges in clinical application

Circulating circRNAs have a higher stability than other RNAs due to their circular structure and critical regulatory role in tumor development, making them a rising star in tumor detection. However, there are still some challenges and problems that need to be addressed in practical clinical application. First, the source of the samples. The samples collected in different studies varied widely, with some are exosomal circRNAs and others are cell-free circRNAs (including free circRNAs that bind with proteins and circRNAs in exosomes or microparticles). The difference in the samples may lead to difference in circRNA content and thus in the origin. In addition, some studies used serum and some used plasma. Serum and plasma have different sensitivities and specificities in tumor detection, and the RNAs they contain are also different (173, 174). The choice of sample will directly affect the type and content of circRNA. The second is the absence of standardized protocols for sample preparation, such as which anticoagulant should be chosen and at what temperature the sample should be stored. In addition, the accidental release of nucleic acid from peripheral blood cells should be fully considered (175, 176). Third, there is the problem of technical methods. The level of circulating circRNAs is extremely low compared to tissue circRNAs. Traditional experimental methods such as PCR, microarray technique and northern blot are no longer suitable for clinical use due to their low specificity and sensitivity. RNA sequencing (RNA-seq) has been widely used for the discovery and quantification of circRNAs. Despite the tremendous successes of short-read RNA-seq studies of circRNAs (177, 178), there are inherent limitations of this approach, especially the inability to capture the full length of circRNA completely and internal alternative splicing events within circRNAs, and the difficulty in accurately quantification of circRNA (179). Long-read RNA-seq (third-generation RNA-seq) technology is a newly developed transcriptome analysis technology (such as the Pacific biosciences (PacBio) and the Oxford nanopore technology company (permanent)), which can produce thousands to tens of thousands of bases in length (180), and can fundamentally eliminate the excessive fragmentation of short-read RNA-seq, bringing hope for full-length detection and accurate quantification of circRNAs. However, it has a higher error rate and lower throughput (181). Although the development of some technologies has made up for the loopholes of the third generation RNA-seq to some extent (181, 182), a technical means with high sensitivity, accuracy, repeatability and universality is still needed in the practical clinical application.

## Conclusion and future prospect

**Figure 1:**
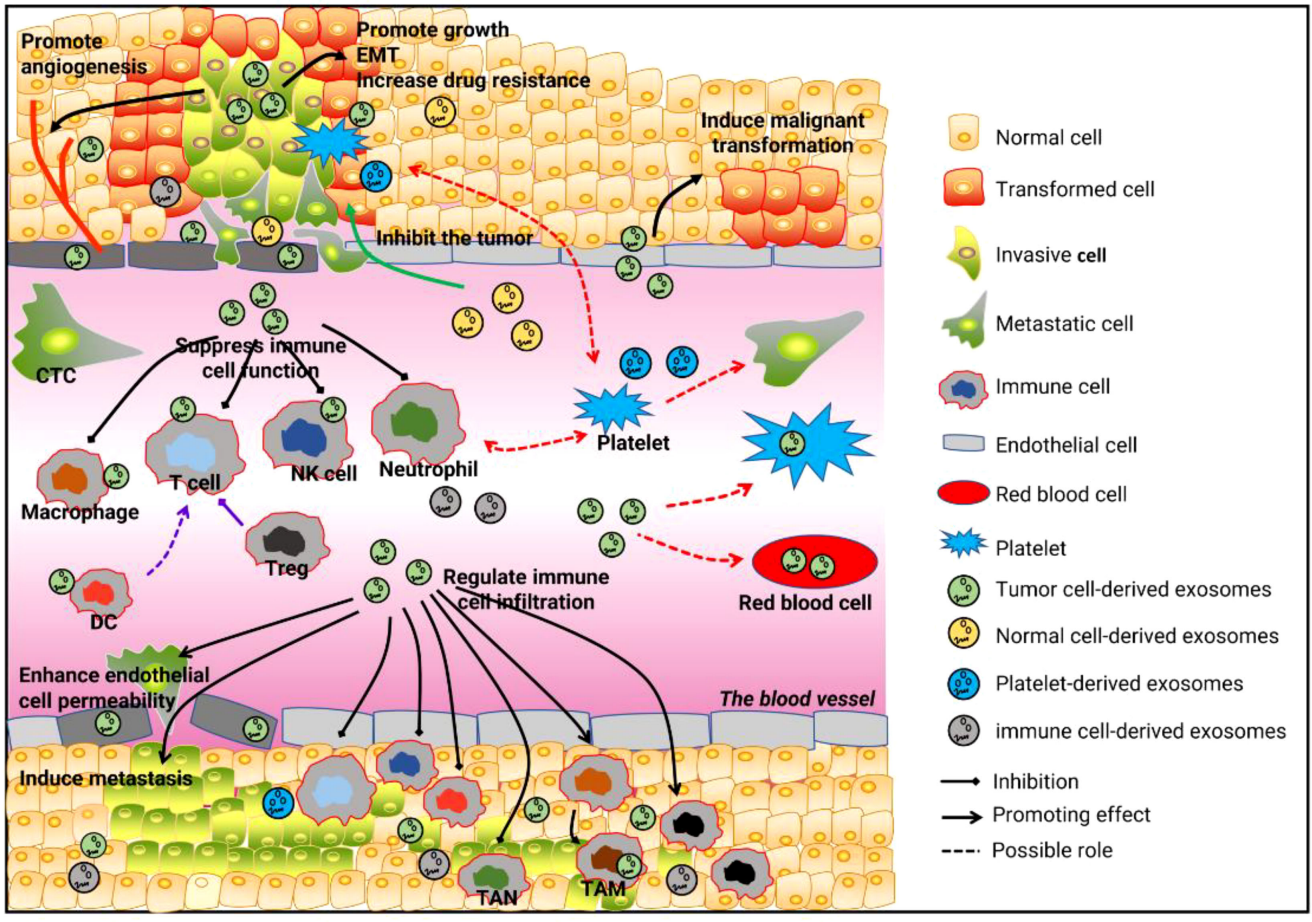
Cell-cell communication is mediated by circulating RNAs and tumor development. First, circulating cirRNAs affect tumor growth, metastasis, angiogenesis, and drug resistance by mediating communication between tumor cells and tumor cells, tumor cells and normal cells, and tumor cells and endothelial cells. Second, circulating cirRNAs mediate communication between tumor cells and immune cells, by which they suppress tumor-related immunity and reshape the tumor microenvironment. Third, the communication between tumor cells and platelets, and between tumor cells and red blood cells, may be involved in the occurrence and development of tumors.

Cancer is a rapidly progressive malignant disease, especially in its terminal stages, that can involve multiple systems and organs in the body. The exchange of information between tissues or cells underlies a range of processes and is key to the occurrence and rapid development of tumors. Due to their unique advantages, circulating circRNAs participate in multiple cell-cell communications and regulate the occurrence and development of tumors from many aspects, which gives them great value and significance in tumor detection and treatment. However, intercellular communication is diverse and complex, and many questions remain open, such as whether circulating circRNAs are involved in the communication between tumor cells and red blood cells, and whether tumor-associated anemia is related to circulating circRNAs. It is also unclear whether tumor-associated thrombosis is associated with platelet uptake of tumor-derived circRNAs (**Figure 1**). In addition, most of the MSCs and immune cells in the tumor microenvironment come from the circulatory system. The role and mechanism of circulating circRNAs or tumor-derived circulating RNAs in the recruitment of these cells remains to be investigated further. An in-depth understanding of cell-cell communication mediated by circulating circRNAs will establish a new mechanism of tumor development from point to surface, breaking the one-way relationship in previous studies and improving the accuracy and specificity of tumor diagnosis and enhancing the effectiveness of tumor-targeted therapies.

## Author contributions

SM drafted and wrote the manuscript. QZ design and revised the manuscript. All authors contributed to the article and approved the submitted version.
